# Regeneration of hamstring tendons after anterior cruciate ligament reconstruction

**DOI:** 10.1007/s00167-012-2125-0

**Published:** 2012-07-05

**Authors:** Rob P. A. Janssen, Maria J. F. van der Velden, Huub L. M. Pasmans, Harm A. G. M. Sala

**Affiliations:** 1 Orthopaedic Center Máxima, Máxima Medical Center, Postbus 90052, 5600 PD Eindhoven, The Netherlands; 2 Department of Radiology, Máxima Medical Center, Postbus 90052, 5600 PD Eindhoven, The Netherlands

**Keywords:** Anterior cruciate ligament reconstruction, Hamstring, Semitendinosus, Gracilis, Regeneration, MRI

## Abstract

**Purpose:**

Primary aim of the study was analysis of hamstring tendon regeneration after anterior cruciate ligament reconstruction (ACLR). Secondary aim was analysis of isokinetic muscle strength in relation to hamstring regeneration. The hypothesis was that regeneration of hamstring tendons after ACLR occurs and that regenerated hamstring tendons contribute to isokinetic hamstring strength with regeneration distal to the knee joint line.

**Methods:**

Twenty-two patients scheduled for ACLR underwent prospective MRI analysis of both legs. MRI parameters were tendon regeneration and morphology, muscle retraction and muscle cross-sectional area. A double-blind, prospective analysis of isokinetic quadriceps and hamstrings strength was performed.

**Results:**

Regeneration of the gracilis tendon after ACLR occurred in all patients. Regeneration of the semitendinosus tendon occurred in 14 patients. At 1 year, the surface area of the semitendinosus and gracilis muscle decreased compared to both preoperatively (*P* < 0.01) and the contralateral leg (*P* < 0.01). The cross-sectional area of the semitendinosus muscle decreased in the absence of tendon regeneration (*P* = 0.05). The cross-sectional area of the gracilis muscle was greater in case of regeneration distal to the joint line (*P* = 0.01). Muscle retraction of the semitendinosus muscle was increased in case of nonregeneration (*P* = 0.02). There was no significant relationship between isokinetic flexion strength and tendon regeneration.

**Conclusion:**

Hamstring tendons regenerated after harvest of both semitendinosus and gracilis tendons for ACLR. There was no relation between isokinetic flexion strength and tendon regeneration.

**Level of evidence:**

Prognostic study, Level II.

## Introduction

Hamstring tendons are frequently used as autograft for single- and double-bundle anterior cruciate ligament (ACL) reconstruction. Regeneration of hamstring tendons, to various extends, has previously been reported [4–7, 14, 16, 20–22, 27]. In 1982, Lipscomb et al. [12] reported results of hamstring muscle strength after ACL reconstruction using autograft hamstring tendons. Regeneration of hamstring tendons after ACL reconstruction was first described by Cross et al. in 1992. Part of their study was analysis of upper leg flexion and extension muscle strength in analogy of the work of Lipscomb et al. [12]. In addition to hamstring regeneration and muscle strength after ACL reconstruction, Simonian et al. [21] examined the cross-sectional area of individual hamstring muscles as well as the insertion site of the regenerated tendons. Later, mostly, retrospective research on regeneration of hamstring tendons after ACL reconstruction focused on muscle cross-sectional area [5, 6, 20, 22, 27], retraction of hamstring muscle [5, 14, 27] and muscle strength [5, 14, 22, 23]. A prospective MRI study, comparing patients with and without tendon regeneration in regard to isokinetic muscle strength, has only been performed by Eriksson et al. [5]. They used a single hamstring tendon (semitendinosus) for ACL reconstruction. To our knowledge, no such study has been performed after harvest of both semitendinosus and gracilis tendons for ACL reconstruction. The primary purpose of the present study was to demonstrate regeneration and morphology of semitendinosus and gracilis tendons after ACL reconstruction using both hamstring tendons. In addition, isokinetic flexion and extension strength were examined between patients with and without hamstring tendon regeneration. The hypothesis was that both semitendinosus and gracilis tendons regenerate after harvest for ACL reconstruction.

Furthermore, we hypothesized that isokinetic muscle strength is better restored in case of regeneration of hamstring tendons distal to the joint line.

## Materials and methods

Inclusion criteria were the following: chronic unilateral ACL-deficient knee without any concomitant knee ligament injury and informed consent to participate in the study.

Exclusion criteria were the following: (1) Fractures of either lower extremity in the past; (2) Previous ACL, hamstring or quadriceps surgery; (3) Contra-indications for MRI.

The study consisted of 2 parts. The first part was a prospective MRI study to determine the regeneration of semitendinosus and gracilis tendons after ACL reconstruction. Consecutive patients, who fulfilled the entry criteria as defined by the study protocol, underwent MRI of both legs preoperatively as well as 2 weeks, 6 and 12 months postoperatively.

All patients were operated by the same surgeon (HS). ACL reconstruction was performed using a quadruple hamstring autograft (semitendinosus and gracilis). All patients were rehabilitated according to a standardized accelerated brace-free rehabilitation programme.

The second part was a prospective, double-blind study of isokinetic strength of quadriceps and hamstring muscles of both legs. Patients were examined preoperatively and 6 and 12 months postoperatively. Patients were evaluated by Tegner, Lysholm and International Knee Documentation Committee (IKDC) scores. Upper leg circumference measurements and KT-1000 laxity testing at 89 and 134 Newton (MEDmetric Co., San Diego, CA, USA) of both legs were performed. An isokinetic strength protocol was used to test quadriceps and hamstring muscles. All patients were examined by the same independent examiner (RJ). Patients and examiner were blinded for the MRI results.

In order to compare the results between patients with and without hamstring regeneration, patients were classified in one of the following 3 groups: no hamstring regeneration, regeneration of one tendon or regeneration of 2 tendons. The last 2 groups (with hamstring regeneration) were further classified in either proximal or distal reattachment of the tendon, in reference to the knee joint line.

The semitendinosus and gracilis muscles have also been divided into separate groups with or without tendon regeneration. In case of tendon regeneration, further subdivision was made in either proximal or distal reattachment of the tendon (in reference to the knee joint line).

Written informed consent was documented in all patients. They participated voluntarily in the study and did not receive a reward of any kind. The study was approved by an independent medical ethics committee (METC-number 0110; Máxima Medical Center, Veldhoven, The Netherlands).

### Surgical procedure

One orthopaedic surgeon (HS) performed ACL reconstruction in all 22 patients. A 4- to 5-cm vertical skin incision was made over the pes anserinus. The crural fascia (layer 1 according to Warren and Marshall [26]) was incised in a longitudinal fashion, proximal to the hamstring tendons extending distally over the pes anserinus. A closed tendon stripper was used to remove the gracilis and semitendinosus tendons. Arthroscopic ACL reconstruction was performed with Bone Mulch screw femoral fixation and WasherLoc tibial fixation (Arthrotek, Warsaw, In, USA). The crural fascia was not sutured. The standardized rehabilitation protocol has been described in a previous publication [10].

### MRI

Preoperatively, MRI scans of both legs were made simultaneously by a standardized protocol with a 1.5 Tesla MRI (Philips Medical Systems, Best, The Netherlands). The MRI of both legs was repeated postoperatively at 14 (~14.3 ± 1.9) days, 6 (~6.2 ± 0.9) months and 12 (~12.4 ± 1.6) months. Both knees were positioned in a body coil in full extension and 15° exorotation. T1- and T2-weighted transaxial images were made starting 1 cm distal to the tibial tuberosity till 30 cm proximal to the knee joint line. Repetition time (TR) was 489s and 2,770 ms, and echo time (TE) was 10 and 100 ms for the T1- and T2-weighted images, respectively. Matrix size was 512 × 512 pixels, and field of view (FOV) was 360 × 360 mm. The slice thickness was 8.0 mm and slice intersection gap 1.0 mm. Sagittal images were also made extending from 8 cm distal to the knee joint line up to 32 cm proximal to the knee joint line. These images were made with TR 500 ms, TE 10 ms, matrix size 512 × 512 pixels, FOV 400 × 400 mm, slice thickness 4.0 mm and slice intersection gap of 0.4 mm.

Measurements were made of the following muscles: semitendinosus, gracilis, semimembranosus and long head of the biceps femoris. On the preoperative scans, the maximal cross-sectional area of all four muscles was measured. The exact distance in the sagittal plane between the joint line and the maximal cross-sectional area was recorded per patient and per muscle. This same distance was used on the postoperative scans of the patient to compare the cross-sectional areas of the four muscles.

Additional MRI parameters determined were the following: (1) distance between the joint line and the preoperative distal muscle-tendon junction of semitendinosus and gracilis; (2) distance between the joint line and the distal muscle ends of semitendinosus and gracilis after tendon harvesting; (3) distance between the joint line and the distal muscle-tendon junction in case of tendon regeneration; (4) distance between the joint line and the distal muscle end in case of no tendon regeneration; (5) anatomic insertion site of regenerated tendon.

The MRI scans were examined by two independent examiners (HP, MV) with measurements made on both legs.

### Isokinetic testing protocol

All patients underwent isokinetic strength test of quadriceps and hamstrings of both legs preoperatively. A standardized test protocol was performed using the Biodex System III dynamometer (Biodex Medical Systems, Shirley, NY, USA). The test protocol was repeated at 6 (~6.6 ± 1.0) months and 12 (~13.0 ± 2.0) months postoperatively. All tests were performed by an independent examiner (RJ). Both examiner and patients were blinded for the MRI results. After a 10-min warm-up period on a cycle ergometer (50 W), isokinetic testing was performed in sitting position with hip flexed 60°. The upper body, pelvis and thigh of the tested upper leg were stabilized with straps. The lower leg fixation was at 20 cm distal to the knee joint line, to minimize the effect of knee joint instability on muscle strength performance. A concentric-concentric knee test protocol, with gravitational correction, was performed allowing full range of flexion and extension at 60, 180 and 300°/s. The patient was instructed to maximally extend the knee (up to the level of the examiners hand) as well as maximal flexion. The test consisted of 5 maximal torques for quadriceps and hamstrings strength at 60, 180 and 300°/s with a 10-s pause between the 3 angle velocities. During 1-min rest, the dynamometer was installed for the contralateral leg and the same test sequence performed. Peak values in Newton meters and total work in Joules (area under the curve) were calculated in each test. The reliability of the test was determined by the variation coefficient and curve pattern. Comparisons between both legs were made as well as comparison in time for each leg separately.

### Statistical analysis

Statistical analysis was performed using SPSS 19.0. A sample size calculation was performed for the study. The primary endpoint was the difference in cross-sectional area preoperatively compared to postoperatively. With a difference of 2 in the mean response and a standard deviation of 3, 20 pairs of subjects were needed. The used alpha associated with this paired test was 0.05, and the power was 0.8. Twenty-two patients were included in the study. The results displayed few normal distributions. For this reason, median instead of average values were used. The Wilcoxon signed rank test was used to determine pre- and postoperative differences as well as the differences between both legs. Differences between patients with and without hamstring regeneration were assessed by the Mann–Whitney *U* test. Significance was set at ≤0.05.

## Results

Twenty-two consecutive patients, who fulfilled the entry criteria as defined by the study protocol, were included in the study. There were 17 men and 5 women with a mean age of 28.4 years ± 5.0 (21–37).

### MRI

A total of 5 out of 88 MRI scans were missing upon review: one preoperative scan, one 2-week postoperative scan, one 6-month postoperative scan and two 12-month postoperative scans. As a consequence, it was not possible to analyse the results of muscle retraction and cross-sectional area in three patients when comparing preoperative and 12-month postoperative results. However, the hamstring regeneration could be evaluated in all patients using either 6- or 12-month postoperative MRI scan.

**Fig. 1:**
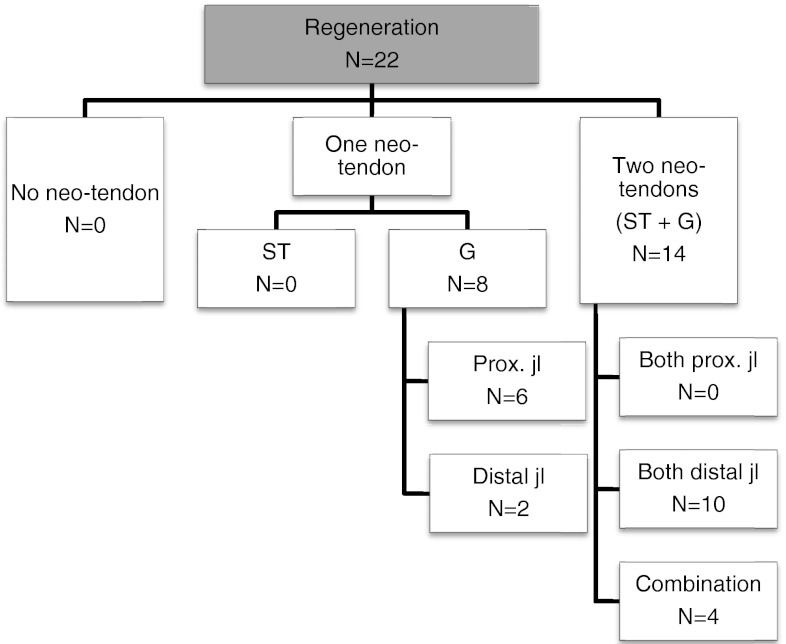
Regeneration of hamstring tendons and the insertion level (*ST* semitendinosus tendon, *G* gracilis tendon, *neo-tendon* regenerated tendon, *prox.* proximal, *jl* joint line)

**Fig. 2:**
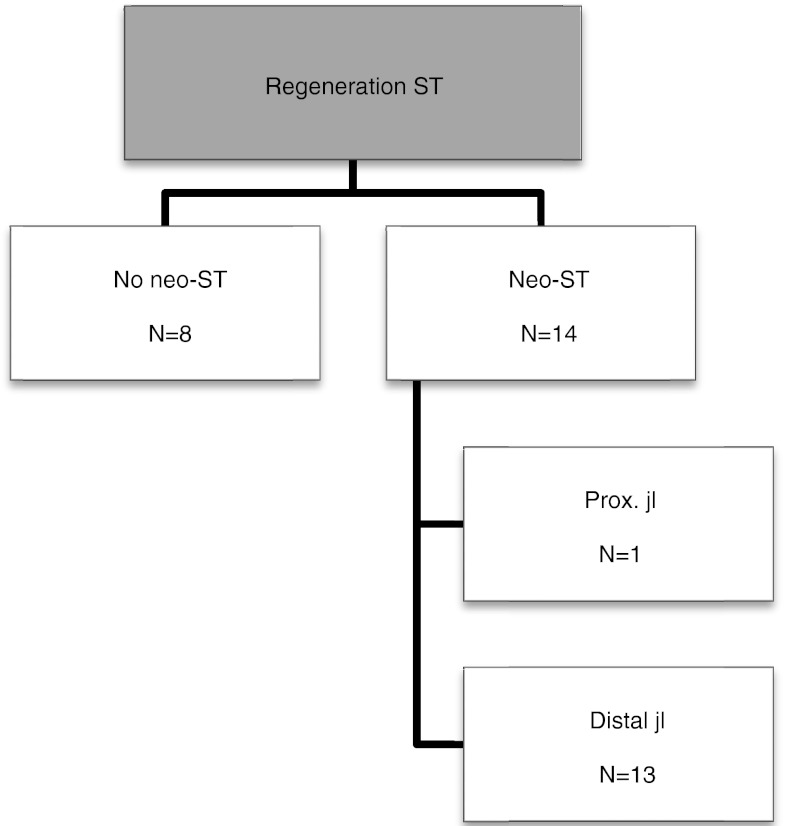
Regeneration of semitendinosus tendon (ST) and the insertion level (*neo-ST* regenerated semitendinosus tendon, *prox.* proximal, *jl* joint line)

**Fig. 3:**
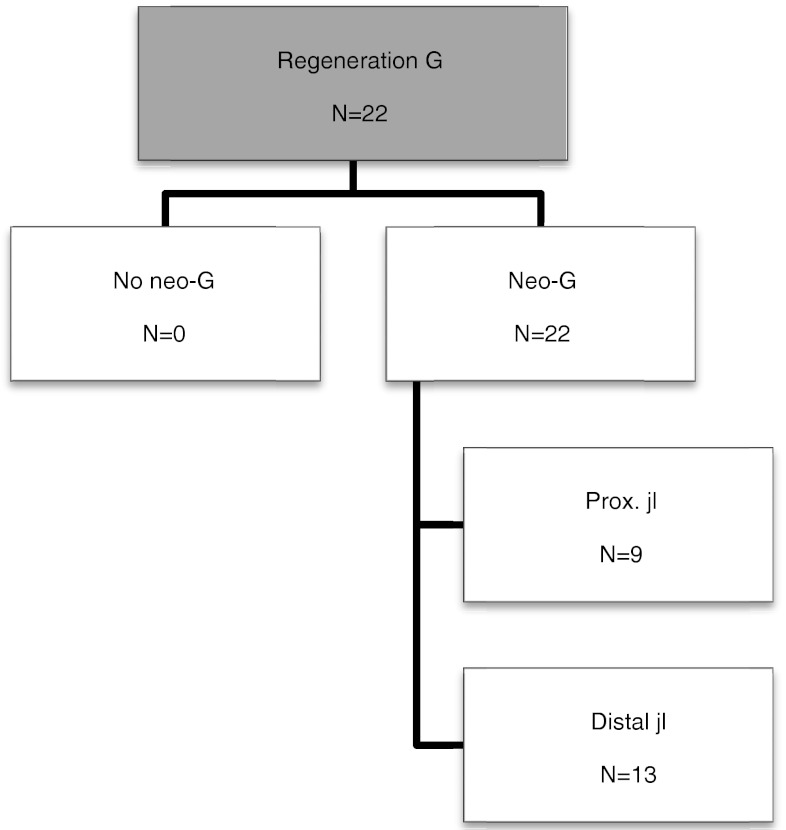
Regeneration of gracilis tendon (G) and the insertion level (*neo-G* regenerated gracilis tendon *prox.* proximal, *jl* joint line)

**Fig. 4:**
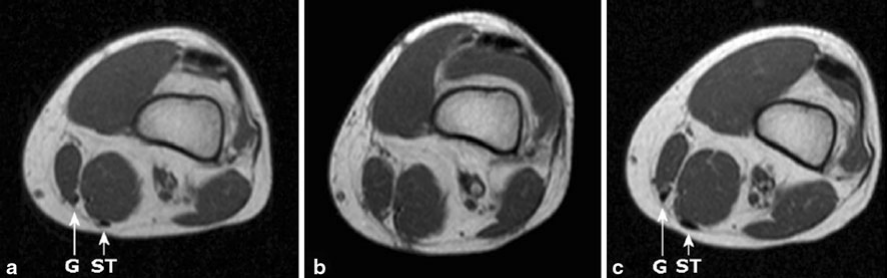
Transverse MRI images of gracilis (G) and semitendinosus (ST) tendons of same patient 6.3 cm proximal to the joint line at time intervals: **a** preoperative; **b** 2 weeks postoperatively; **c** 12 months postoperatively

**Fig. 5:**
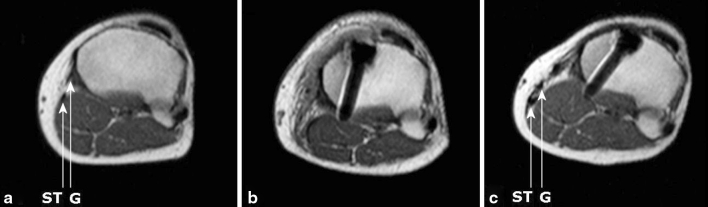
Transverse MRI images of gracilis (G) and semitendinosus (ST) tendons of same patient 2.7 cm distal to the joint line at time intervals: **a** preoperative; **b** 2 weeks postoperatively; **c** 12 months postoperatively

The results of hamstring regeneration are presented in Fig. 1. All 22 patients demonstrated hamstring regeneration after harvest for ACL reconstruction. Figures 2 and 3 show the specific results of semitendinosus and gracilis regeneration, respectively. Figures 4 and 5 demonstrate a series of MRI proximal and distal to the joint line in a patient with regeneration of both semitendinosus and gracilis tendons. Results of cross-sectional area of semitendinosus and gracilis muscles are presented in Tables 1, 2, 3 and 4. All gracilis tendons regenerated. For that reason, the gracilis muscle cross-sectional area in the group of patients with tendon regeneration proximal to the joint line was compared to the group of patients with gracilis regeneration distal to the joint line (Table 4). Table 5 demonstrates the amount of retraction of semitendinosus muscles. There was no significant compensatory hypertrophy of the semimembranosus and biceps femoris muscles after hamstring tendon harvest.

**Table 1:** Cross-sectional area (cm^2^) of the semitendinosus and gracilis muscles preoperatively and at 12 months postoperatively

	Preoperative	12 months postoperatively	*P* value
Semitendinosus	12.2 (±3.3)	8.3 (±3.0)	<0.01
Gracilis	4.9 (±1.2)	3.6 (±1.2)	<0.01

**Table 2:** Cross-sectional area (cm^2^) of the semitendinosus and gracilis muscles comparing the operated versus the contralateral leg at 12 months postoperatively

	Operated leg	Contralateral leg	*P* value
Semitendinosus	8.3 (±3.0)	14.0 (±4.1)	<0.01
Gracilis	3.6 (±1.2)	5.1 (±1.4)	<0.01

**Table 3:** Cross-sectional area (cm^2^) of the semitendinosus muscles without tendon regeneration and regeneration distal to the joint line at 12 months postoperatively (neo-tendon, regenerated tendon)

	No neo-tendon	Neo-tendon distal to joint line	*P* value
Semitendinosus	6.0 (±2.1)	10.0 (±2.6)	0.05

**Table 4:** Cross-sectional area (cm^2^) of the gracilis muscles with tendon regeneration proximal and distal to the joint line at 12 months postoperatively (neo-tendon, regenerated tendon)

	Neo-tendon proximal to joint line	Neo-tendon distal to joint line	*P* value
Gracilis	2.8 (±0.7)	4.8 (±1.1)	0.01

**Table 5:** Semitendinosus muscle retraction (cm) without tendon regeneration and regeneration distal to the joint line at 12 months postoperatively (neo-tendon: regenerated tendon)

	No neo-tendon	Neo-tendon distal to joint line	*P* value
Semitendinosus	13.0 (±3.4)	3.8 (±2.0)	0.02

### Clinical outcome and isokinetic strength

Sixteen of the 22 patients have been evaluated at clinical and isokinetic follow-up at 12 months postoperatively. The remaining group of 6 patients was evaluated at 6 months postoperatively only. They did not return for follow-up at 12 months. The rehabilitation was not considered complete at 6 months postsurgery; therefore, these 6 patients were not included in the final review of clinical outcome and isokinetic strength analysis. The clinical outcomes are presented in Table 6.

**Table 6:** Clinical outcomes

	Preoperative	12-months postoperative	*P* value
*IKDC*			
A	0 (0 %)	2 (13 %)	
B	0 (0 %)	10 (62 %)	
C	4 (18 %)	3 (19 %)	
D	18 (82 %)	1 (6 %)	
Tegner	4 (3–5)	7 (4–9)	<0.01
Lysholm	70 (±10) points	93 (±10) points	<0.01
*KT*-*1000 side to side difference*			
89 N	5 (±3) mm	2 (±4) mm	<0.01
133 N	7 (±3) mm	2 (±3) mm	<0.01
*Upper leg circumference*			
Operated leg	40 (±3) cm	39 (±3) cm	0.05
Contralateral leg	40 (±2) cm	40 (±2) cm	

No significant differences were found when comparing pre- and postoperative isokinetic extension and flexion strength in terms of: (1) peak torque and total work between the operated and contralateral leg; (2) percentage increase or decrease in peak torque and total work between the operated and contralateral leg. No significant differences were found in flexion and extension strength (peak torque and total work) between the group of patients with regeneration of both hamstring tendons distal to the joint line and the group of patients with only 1 regenerated tendon proximal to the joint line.

## Discussion

The most important finding of the present study was that hamstring tendons regenerate after ACL reconstruction. There was no relation between isokinetic flexion strength and tendon regeneration.

Regeneration of all gracilis tendons after ACL reconstruction, with harvest of both gracilis and semitendinosus tendon, was found in MRI studies by Simonian et al. [21] and Williams et al. [27]. Williams et al. found 63 % of the regenerated gracilis tendons to insert distal to the joint line, Simonian et al. 33 %. Regeneration of the semitendinosus tendon occurred in 7 out of 8 patients (88 %) in the study by Williams et al. [27] They described that 25 % attached distal to the joint line. Simonian et al. [21] described semitendinosus tendon regeneration in 6 out of 9 patients (66 %); all 6 tendons inserted on the tibia. These results are similar to the results of the present study. In contrast, Takeda et al. described a group of 11 patients with semitendinosus regeneration in all cases. In 10 patients (91 %), the semitendinosus tendon inserted distal to the joint line. The gracilis tendon regenerated in 9 of their patients (82 %), but none inserted on the tibia [22]. Tadokoro et al. examined a larger group of 28 patients. They described a 79 % semitendinosus tendon regeneration with only 46 % of gracilis tendon regeneration. The authors did not specify the level of insertion [23]. In their MRI follow-up study following hamstring harvest for ACL reconstruction, Rispoli et al. [20] did not make a distinction between semitendinosus and gracilis tendon regeneration.

Various theories exist to explain the phenomenon of regeneration of hamstring tendons after harvest for ACL reconstruction. Some authors [4, 20] postulated regeneration to start at the distal end of semitendinosus and gracilis muscle for reason of increased vascularity. The tendon then regenerates in a distal fashion. Cross et al. [4] and Rispoli et al. [20] viewed the anatomic space between medial layer 1 and 2 [26] as a tubular pathway for the regenerating tendons. This is in analogy of repair of nerve lesions along intact epineural tissue [4, 20]. Tadokoro et al. hypothesized that the gracilis tendon is surrounded by less fascial layers than the semitendinosus tendon. They reported this as a possible explanation for their results of less gracilis tendon regenerations compared to semitendinosus tendon regenerations [23]. This theory of regeneration is not supported by the work by Simonian et al. [21], Williams et al. [27], as well as the present study where gracilis tendon regeneration occurred more frequently than semitendinosus tendon regeneration. Carofino et al. also opposed to this theory. In contrast to the view of Cross et al. [4] and Rispoli et al. [20], Carofino et al. described the pathway between medial layer 1 and 2 as not being tubular in shape. For this reason, they concluded that these fascial layers cannot lead to the similar shape of the regenerated tendons compared to their original morphology [3].

Other authors postulated a second theory to explain hamstring regeneration after harvest for ACL reconstruction. In the void space following harvest, a haematoma is formed. Fibroblast precursor cells migrate from surrounding tissues into this haematoma. They start fibroblast proliferation and collagen production. Limited mechanical stress leads to organization of collagen fibres and possible maturation into a regenerated hamstring tendon [7, 19].

Histological studies of regenerate tendons have found very similar tissue compared to the original hamstring tendons [8, 18]. At 1-year follow-up, the regenerated tendon showed longitudinal, well-organized collagen with fibroblast-like nuclei. However, the total distribution of collagen fibres and cell nuclei was more irregular in comparison with the original tendon tissue [18]. At 2-year follow-up, the central zone of the regenerated tendon demonstrated collagen fibre bundles surrounded by fibrous tissue with fibroblast proliferation [8].

Previous studies on hamstring tendon regeneration, in relation to morphology and/or muscle strength, did not distinguish between patients with or without tendon regeneration [4, 17, 20–23, 27]. In the present study, an analysis was made of muscle retraction, cross-sectional area and isokinetic flexion strength comparing patients with or without tendon regeneration. All patients showed a significant decrease in muscle cross-sectional area after 12 months for both semitendinosus and gracilis muscle, in comparison with preoperative and contralateral values. Similar results were found by other authors. Williams et al. reported a significant decrease in muscle cross-sectional area as well as muscle volume of both semitendinosus and gracilis muscles at 6 months postsurgery in comparison with the preoperative and contralateral values [27]. At 12–16 months postsurgery, Irie et al. found a decrease in muscle cross-sectional area of 47.1 % for semitendinosus and 51.1 % for gracilis muscles compared to the contralateral leg [9]. Eriksson et al. [5], Makihara et al. [13] and Nishino et al. [17] found a significant decrease in semitendinosus cross-sectional area compared to the contralateral leg. In their cases, only the semitendinosus tendon was harvested for ACL reconstruction. In contrast, Rispoli et al. [20] and Takeda et al. [22] reported no significant decrease in semitendinosus cross-sectional area after ACL reconstruction using both semitendinosus and gracilis tendons. However, regarding the gracilis cross-section area, Takeda et al. [22] did report a significant decrease in cross-sectional area. In the present study, a significant decrease in cross-sectional area for both semitendinosus and gracilis was demonstrated regardless of tendon regeneration. In addition, the cross-sectional area of both semitendinosus and gracilis muscles was significantly smaller in case of regeneration of both tendons distal to the joint line compared to regeneration of only one tendon proximal to the joint line. This would suggest that tendon regeneration, distal to the joint line, leads to a more functional muscle condition. Eriksson et al. [6] found similar significant results. In contrast to the present study, they only harvested the semitendinosus tendon for ACL reconstruction.

Hypothetically, the amount of compensatory hypertrophy of semimembranosus and biceps femoris muscles may be related to the number of harvested hamstring tendons for ACL reconstruction. Eriksson et al. [5] demonstrated this phenomenon in patients without regeneration of the harvested semitendinosus tendon. In contrast, the present study did not show significant compensatory hypertrophy of semimembranosus nor biceps femoris muscles after harvest of both gracilis and semitendinosus tendons for ACL reconstruction. These results are similar to the findings by Simonian et al. [21] and Takeda et al. [22].

Nakamae et al. [14], Nishino et al. [17] and Williams et al. [27] found significant muscle retraction of both hamstring tendons after ACL reconstruction. Similar results were found in the present study. If a tendon does not regenerate after harvest, the muscle appears to be nonfunctional as demonstrated by the progressive muscle retraction up to 1 year postsurgery.

No relation was found in the present study between regeneration of hamstring tendons after ACL reconstruction and isokinetic flexion and extension muscle strength. Eriksson et al. [5] and Tadokoro et al. [23] similarly found no significant difference in muscle strength between patients with and without tendon regeneration. Kim et al. performed a comparative study between hamstring-harvested and hamstring-unharvested patients after ACL reconstruction. They showed a significant knee flexion weakness in the operated leg compared to the contralateral side, regardless of hamstring harvesting [11].

There are some limitations to the present study. The isokinetic strength testing did not include deep flexion and internal rotation of the tibia. Various studies have demonstrated that these two factors may be significantly decreased after ACL reconstruction with hamstring tendons [1, 2, 9, 13, 15, 23–25]. It cannot be ruled out that these specific muscle strengths could have shown a significant decrease in patients without tendon regeneration in the present study. The second limitation to the present study is the absent follow-up of 6 patients for isokinetic testing at 12 months postoperatively. This has reduced the number of patients to 16 (out of 22) in the isokinetic strength analysis.

The clinical relevance of the present study is that patients may be informed that hamstring tendons regenerate after retrieval for ACL reconstruction. It also indicates that hamstring autograft ACL reconstruction may be associated with less morbidity than previously thought. This might influence future rehabilitation protocols.

## Conclusion

Hamstring tendons regenerated after harvest of both semitendinosus and gracilis tendons for ACL reconstruction. There was no correlation between isokinetic flexion strength and tendon regeneration.
